# Intramolecular Hydrogen-Bond Activation: Strategies, Benefits, and Influence in Catalysis

**DOI:** 10.1021/acsorginorgau.1c00053

**Published:** 2022-02-03

**Authors:** Andrea Guerrero-Corella, Alberto Fraile, José Alemán

**Affiliations:** † Organic Chemistry Department, Módulo 1, Universidad Autónoma de Madrid, 28049 Madrid, Spain; ‡ Institute for Advanced Research in Chemical Sciences (IAdChem), Universidad Autónoma de Madrid, 28049 Madrid, Spain

**Keywords:** intramolecular activation, hydrogen bonding, organocatalysis, metal catalysis, bifunctional catalysis, asymmetric synthesis

## Abstract

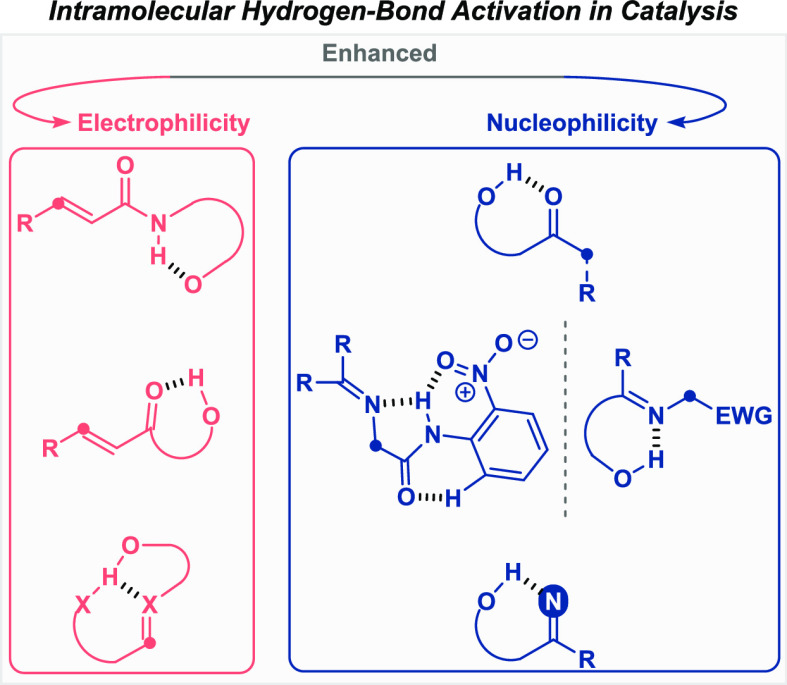

The activation of molecules through intramolecular hydrogen-bond formation to promote chemical reactions appears as a suitable strategy in organic synthesis, especially for the preparation of chiral compounds under metal and organocatalytic conditions. The use of this interaction has enabled reactivity enhancement of reagents, as well as stabilization of the chemical species and enantiocontrol of the processes.

Among the large number of strategies for activating molecules, such as thermal, light, or electrochemical methods,^1^ as well as the use of metals,^2,3^ photoredox,^4,5^ or (non)covalent interactions with multiple organic molecules^6−8^ via catalysis, hydrogen bonding has proven to be exceptional for the development of certain chemical reactions throughout the years. The power of this phenomenon has triggered many organic transformations that occasionally demand more activated substrates through inter- or intramolecular H-bond formation. Thus, the former account for the contact between substrates and catalysts, whereas the latter is based on the hydrogen-bond construction in the reagents themselves.

For this H-bond to be formed, chemical structures must have the possibility to establish a noncovalent interaction between a XH-like motif and an electronegative atom (Y), resulting in an XH···Y bond type.^9^ The presence of XH groups in organic compounds, with X = N or O, is commonplace, so this intramolecular interaction seems an easy and straightforward strategy for synthesis. Therefore, it has permitted an increase in reactivity, together with the stabilization and stereoselectivity improvement of structures and asymmetric processes, respectively.

Consequently, carbon atoms taking part in C–C and C–X (with X = O, N) double bonds, methylene and methyl groups, as well as nitrogen atoms in imine derivatives have benefited from this intramolecular activation to enhance their reactivity as nucleophiles or electrophiles in different catalytic reactions (Figure 1, top). In addition, some of these transformations have been involved in the synthetic routes of relevant compounds in medicinal chemistry such as insecticides,^10^ (+)-VNI^11^ and (−)-physostigmine,^12^ among others, as part of intermediate compounds (Figure 1, bottom). On the other hand, given that several functional groups employed for this purpose can be removed from the structure of the final product once the chemical reaction is finished, this mode of activation arises as an excellent strategy with an increased potential for chemists, not only because of the numerous transformations that can be driven but also because of its diversity.

**Figure 1:**
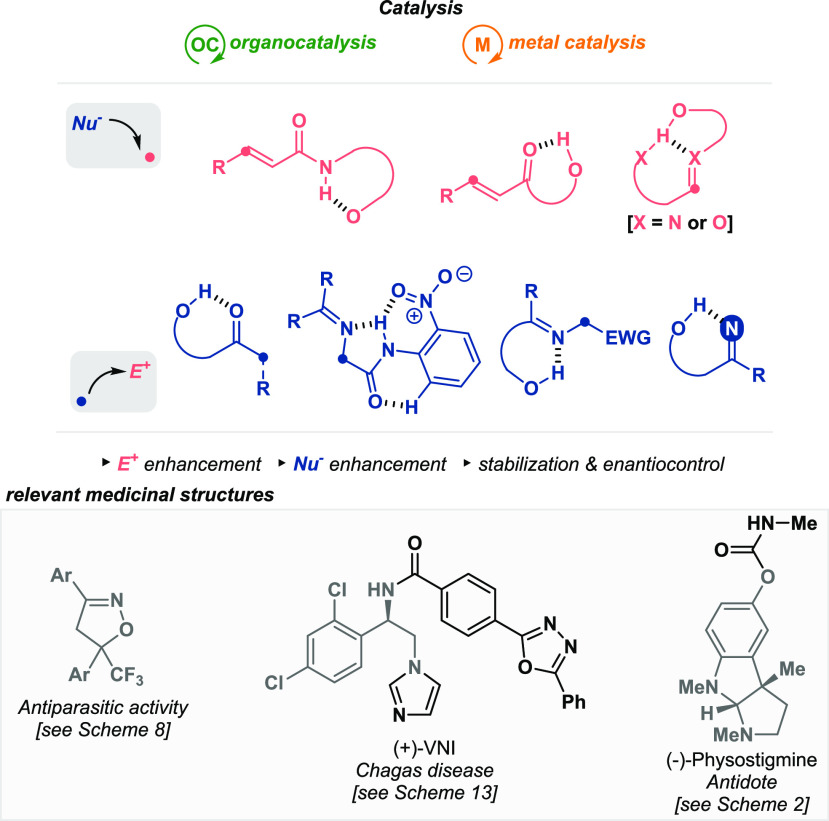
General overview of the activation of substrates in catalysis by intramolecular hydrogen bonding and the potential for organic synthesis.

We hope this Perspective can complement some specific works already published in the literature focused on azomethine ylide derivatives,^13^ providing a general outline on this intramolecular H-bond interaction for the activation of multiple substrates in metal and organocatalytic areas. To do so, different examples from the last 15 years have been reviewed, focusing on functional group types that have been boosted as electrophiles and nucleophiles. In this review, we have only covered those examples in which hydroxyl or amine groups are able to enhance the reactivity and not act like a mere spectator. Furthermore, mechanistic proposals and proofs of concept to emphasize the significant role of this strategy have been detailed. To conclude, the intramolecular role of the hydrogen bond for stabilization and enantiocontrol purposes is also highlighted at the end of this paper.

Over the last years, asymmetric organocatalytic Michael additions and Mannich or aldol reactions have shown some electrophilicity issues for the construction of carbon–carbon bonds that have been resolved by means of this simple interaction, which has enhanced both reactivity and selectivity of the processes. In this context and taking advantage of the way double H-bond motifs such as thioureas coordinate to oxygen atoms, Takemoto established that imides might have a proper structure able to match with a bifunctional organocatalyst via hydrogen bonding (Scheme 1).^14^ Different α,β-unsaturated imides were evaluated in the asymmetric conjugate addition of malononitriles; however, *N*-acylbenzamides with a methoxy group at the *ortho* position of the aryl ring stood out as the greatest substituent for the transformation. Considering the excellent yields and enantiomeric excesses in short reaction times in most of the cases, the authors suggested a double activation of the Michael acceptor due to (i) the intramolecular hydrogen bond between NH and MeO groups, which increases the electrophilicity of the unsaturated carbonyl system, and (ii) the intermolecular hydrogen bonding of the imide with the catalyst that favors enantioselectivity. Furthermore, the development of 1,4-additions with other nucleophiles was also accomplished with excellent results given the bifunctional character of the catalyst.

**Scheme 1:**
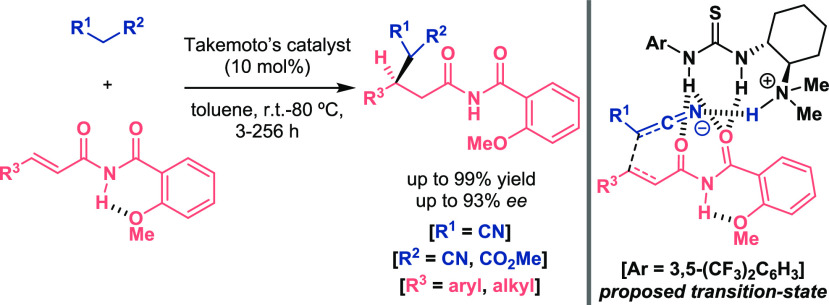
Activation of α,β-Unsaturated Carbonyl Compounds for Enantioselective Organocatalytic Michael Addition Reaction (Takemoto 2006)

Similar to the method used for imides, Palomo and co-workers developed Michael addition reactions using α-hydroxy enones as α,β-unsaturated carboxylic acid surrogates (Scheme 2).^15,16^ These substrates were tested against multiple nucleophiles, namely, 3-substituted oxindoles, α-substituted cyanoacetates, and oxazolones, among others, to demonstrate the generality of the method. Finally, the ketol unit of the final products could be easily transformed into other functional groups such as carboxylic acids, aldehydes, or ketones, giving rise to enantioenriched structures undoubtedly appealing in the synthesis. Density functional theory (DFT) calculations complemented this broad study and explained the exact role of the ketol moiety, which displayed a perfect structure to carry out the transformation since the hydroxyl group established a hydrogen bond with the carbonyl of the enone, leading to a more electrophilic unsaturated functionality.

**Scheme 2:**
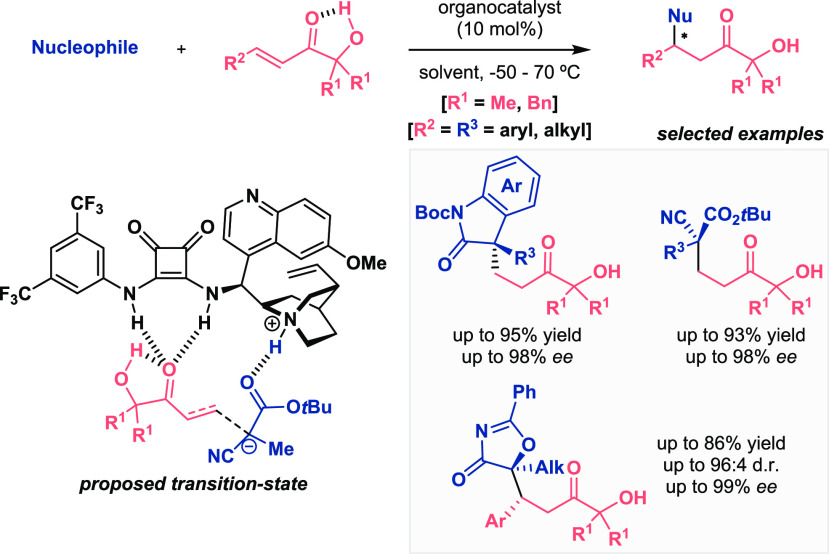
Activation of α,β-Unsaturated Carbonyl Compounds for Enantioselective Organocatalytic Michael Addition Reaction (Palomo 2014 and 2016)

The capture of in situ generated oxonium ylides with imines has been a challenge because two possible routes can be followed once the active intermediate is formed (Scheme 3): (a) nucleophilic addition to the imine to achieve the desired Mannich adduct (colored product) or (b) rearrangement by a 1,2-hydride shift, giving rise to the undesired product (gray product). For the purpose of avoiding this fast latter route, Hu’s group proposed more electrophilic imines with the aim of enhancing the reaction rate toward the nucleophilic addition pathway.^17^ They used aryl imines bearing an *ortho*-hydroxyl group, in which an intramolecular hydrogen bond between the OH group and the iminic nitrogen was formed, thereby activating the C=N bond. Therefore, 3-amino-2-hydroxyesters were synthesized as the main products in a highly diastereoselective fashion. This interaction was demonstrated to be responsible for the imines’ reactivity increase in comparison with how the *N*-phenyl imine afforded the nondesirable product as a major compound. These types of imines were already employed by Akiyama in a classic example in the field of phosphoric acid catalysts;^18^ however, the role of the intramolecular hydrogen-bond interaction was not clearly specified for the addressed purpose given the coordination of the organocatalyst and the imine substrate proposed.

**Scheme 3:**
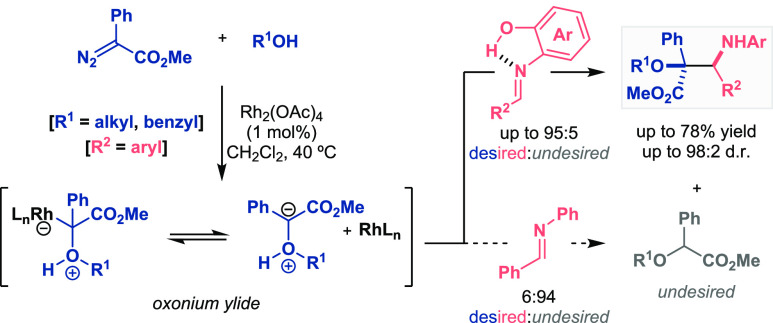
Activation of Imines for Metal-Catalyzed Mannich Reactions (Hu 2007)

In three similar works,^19−21^ Wang demonstrated how a chiral phosphoric acid (CPA) brilliantly catalyzed the enantioselective reduction of aryl ketimine derivatives with Hantzsch esters as a hydride source for the preparation of optically active amines (Scheme 4). They confirmed that the presence of the OH in the aromatic ring permitted the intramolecular hydrogen-bond formation, which conferred (i) stabilization to the imines, (ii) electrophilicity enhancement of the C–N double bond for the hydrogenation, and (iii) improved enantioselectivities due to an appropriate organization between the catalyst and the reagents based on a H-bond network.

**Scheme 4:**
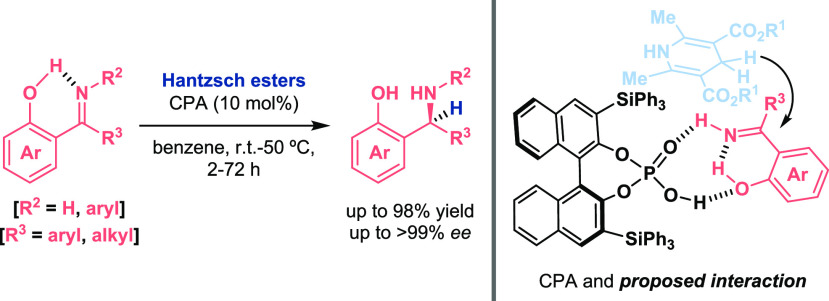
Activation of Imines for Asymmetric Reduction Reactions Catalyzed by a CPA (Wang 2010 and 2011)

Additional activation of the aldimine’s intrinsic mode of reactivity was also necessary when Bolm synthesized optically active *trans*-γ-lactams via N-heterocyclic carbene (NHC) catalysis (Scheme 5, top). Consequently, the corresponding reactive intermediates were efficiently added to the activated OH imines.^22^ In this report, the crucial role of the OH moiety was proven when aldimines without the OH or bearing the protected oxygen atom were tested and no reactivity was observed. An analogous concept has recently been shown by Biju for the asymmetric intramolecular cyclization reaction through umpolung of aldimines catalyzed by a chiral triazolium salt (Scheme 5, bottom).^23^ DFT calculations showed that the aza-Breslow intermediate generated in the media was stabilized due to intramolecular hydrogen-bond interactions from the OH group, triggering nucleophilic addition to the remaining aldimine with enhanced electrophilicity as well by this motif.

**Scheme 5:**
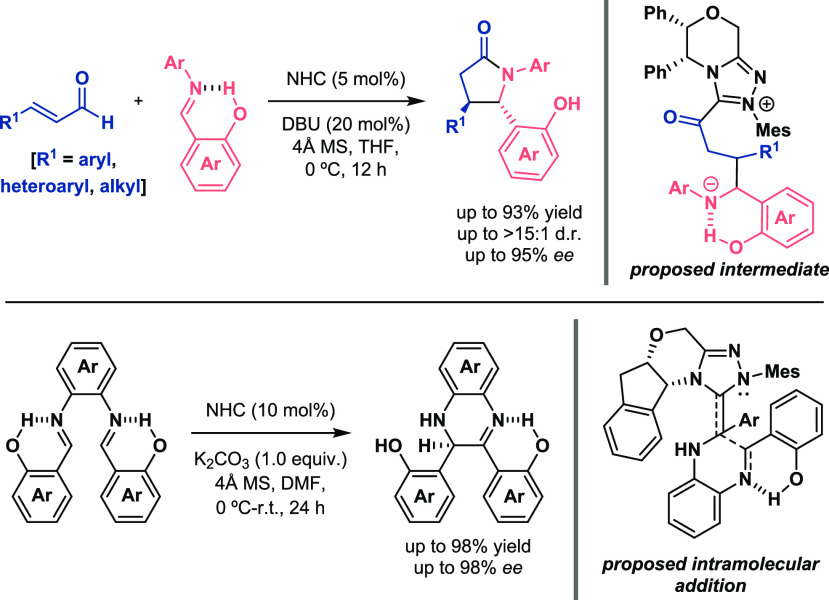
Activation of Aldimines for Enantioselective NHC-Catalyzed Reactions (Bolm 2017, Top, Biju 2019, Bottom)

In addition to imines, aldehydes can also experience this electrophilicity increase to carry out aldol-type transformations. Yang described a phosphine-catalyzed reaction between ynones and benzaldehydes to access furan-3-one derivatives (Scheme 6, top).^24^ The OH and NH groups placed at the *ortho* position of the aromatic ring of the aldehyde established an intramolecular interaction (XH···O), activating the carbonyl group for the nucleophilic attack of the enolate intermediate generated in situ. Regarding asymmetric processes, Krische found that the use of *N*-Boc-α-aminoaldehydes was the key for the stereocontrolled reductive aldol reaction of vinyl ketones to α-chiral aldehydes (Scheme 6, bottom).^25^ The presence of an intramolecular hydrogen bond between CHO···HNBoc was a determinant for the transformation in terms of reactivity (excellent yields) and stereoselectivity (>20:1 value) of the final compounds. The importance of this interaction was demonstrated when the NH moiety was methylated and lower values for the aldol adducts (66 versus 17% yield; 20:1 versus 7:1 dr) were achieved.

**Scheme 6:**
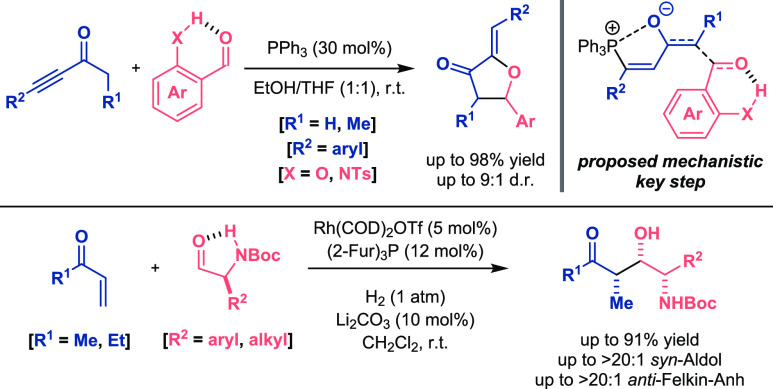
Activation of Aldehydes for Aldol-Type Reactions (Yang 2019, Top, Krische 2006, Bottom)

This phenomenon also assisted the reaction reported by Smith for the construction of enantioenriched pyrroloindoline derivatives under phase-transfer catalyst (PTC) conditions (Scheme 7).^26,27^ First, asymmetric conjugate addition of the deprotonated isocyanide to the α,β-unsaturated ester mediated by a chiral PTC led to the corresponding enolate that underwent subsequent cyclization with the isocyanide group (see Scheme 7, key mechanistic steps). For this step, due to the pretty unusual electronic structure of the isocyanide, an intramolecular H-bond interaction was decisive to drive the first cyclization point. After that, proton transfer followed by a second cyclization concluded the reaction to give the final tricyclic products in good yields and stereoselectivities.

**Scheme 7:**
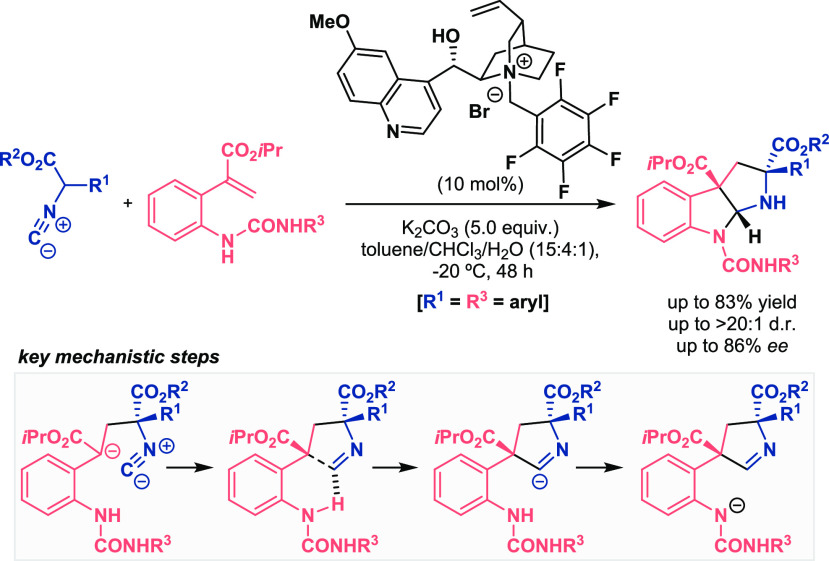
Activation of Isocyanide for Asymmetric PTC-Catalyzed Cyclization Reactions (Smith 2014)

Despite multiple examples of enhanced electrophilicity, the use of this H-bond interaction in the context of nucleophile activation has been less explored. The lack of acidity in some molecules limits certain organic transformations in which a deprotonation step is necessary for the generation of the required nucleophilic carbon. In particular, carbonyl compounds with adjacent hydrogens can suffer enolization processes, but depending on the nature of these carbonyl groups, the acidity of the α protons varies, making the deprotonation step an issue. This problem usually occurs in the asymmetric organocatalytic field owing to the general low basicity of common organocatalysts. Therefore, some researchers have shown the generation of enolates assisted by the formation of an intramolecular hydrogen bond and their subsequent addition to electrophilic counterparts.

Within this context, the use of aromatic ketones as pronucleophiles has been a matter of concern because of their high p*K*_a_ value.^28^ Therefore, their role as a donor species in aldol-type reactions has demanded strong bases for the generation of the corresponding enolates. Hence, Da and co-workers reported the formation of an enolate from the *o*-hydroxyacetophenone due to acidity growth of the hydrogen atoms at the α position due to the intramolecular H-bond present in the aryl ketone (Scheme 8).^29^ Deprotonation by a weak base such as the tertiary amine of a bifunctional organocatalyst promoted the enantioselective addition to trifluoromethyl ketones. The most important feature is the ambivalent role of the OH group, which enhances reactivity and improves selectivity of the process, given that the reaction with acetophenone under the same reaction conditions achieved the final product in 33% yield and only 60% ee. Finally, authors suggested that both reaction counterparts and the bifunctional thiourea gathered in a proper fashion for the nucleophilic attack, achieving the final β-hydroxy-β-trifluoromethyl derivatives in good results.

**Scheme 8:**
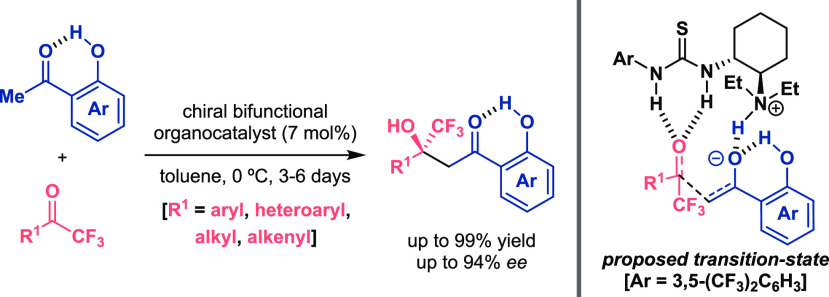
Activation of Ketones for Enantioselective Organocatalytic Direct Cross-Aldol Reactions (Da 2017)

Other carbonyl derivatives can also suffer from acidity problems, which makes their α-functionalization tricky sometimes. The group of Palomo considered the late-stage easy-tunable α-hydroxy ketones as nucleophilic compounds in different catalytic transformations (Scheme 9).^30,31^ The power of the intramolecular hydrogen bonding for the generation of the enol anion was studied in the enantioselective organocatalytic 1,4-addition reaction to nitroalkenes. The presence of the OH group helped in the deprotonation step, so that mild bifunctional catalysts were able to promote the formation of the enolate or formal dienolate. Subsequent α-addition to the nitro-olefin provided access to carbonyl derivatives in excellent yields and diastereo- and enantioselectivities. The exceptional stereochemical outcome was proposed to be conducted by Pápai’s model, in which the double H-bond donor motif coordinated the nucleophilic ketone and the protonated tertiary amine the nitroalkene. Furthermore, the role of the hydroxyl group as the one responsible for the increased acidity was demonstrated when the reaction with a substrate bearing the protected O-TMS reached just 75% conversion in more than 70 h. Moreover, additional straightforward transformations of the ketol moiety into other functional groups such as carboxylic acid or thioester derivatives highlighted the importance of this activating unit (see selected derivatization, Scheme 9, bottom).

**Scheme 9:**
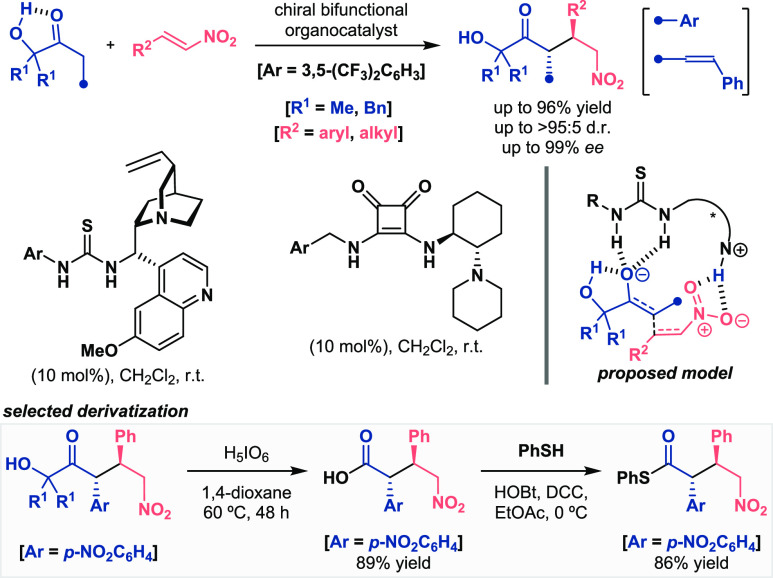
Activation of Ketols for Enantioselective Organocatalytic Michael Addition Reactions (Palomo 2017 and 2018)

In addition, the same research group has recently identified *o*-nitroanilide as an appropriate substituent for improving the reactivity of glycine ketimine derivatives (Scheme 10).^32^ It is well-known the limitations that monoactivated azomethine ylide structures present in asymmetric organocatalytic reactions, in which normally two electron-withdrawing groups α to the nitrogen atom are required,^33,34^ or even the use of highly reactive counterparts^35^ or organosuperbases as catalysts.^36,37^ In this report, they proposed the use of an imine capable of forming three hydrogen-bond intramolecular interactions. This fact resulted in the increased acidity of the methylene hydrogens and the preferred *E*-enolate formation by the organocatalyst (DFT calculations showed the stability of *E-* over *Z*-formation). With this strategy, they developed the aldol reaction between azomethine ylides and aldehydes with excellent results in terms of yields and stereoselectivities. This report appears to be an interesting example for methylene acidity enhancement because the activating group could be removed from the final product in two steps (see selected derivatization, Scheme 10, bottom). To conclude, the authors suggested a hydrogen-bonded system based on inter- and intramolecular interactions between the reagents and the bifunctional organocatalyst to explain the *syn*-selectivity in the final products. Therefore, the coordination of the in situ generated enolate to the urea and the aldehyde to the protonated tertiary amine enabled the nucleophilic attack.

**Scheme 10:**
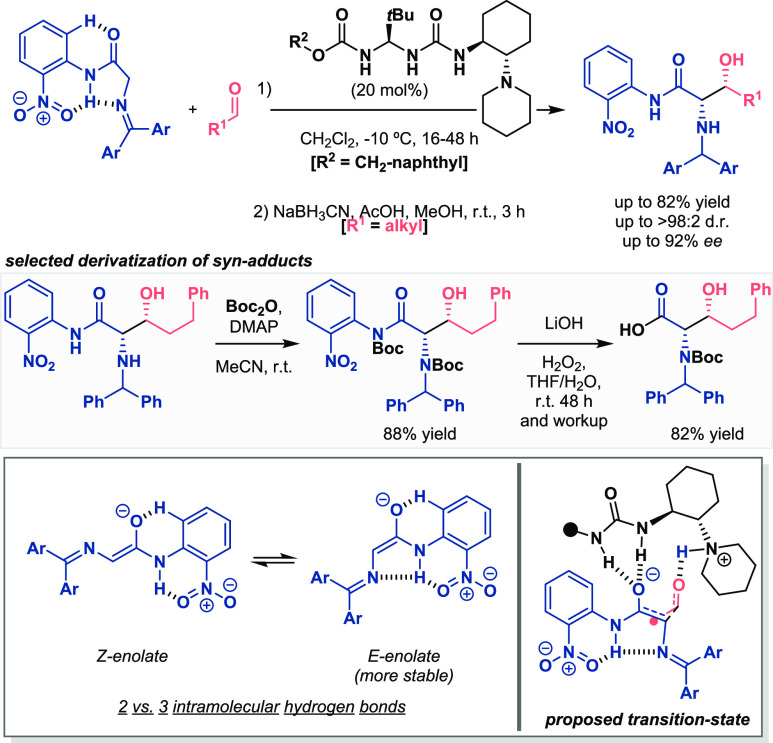
Activation of Methylene Hydrogens for Enantioselective Organocatalytic Aldol Reactions (Palomo 2021)

Our research group has also made a special effort in the activation of C- and N-centered nucleophiles under organocatalytic conditions.^38−41^ The aforementioned acidity issues with respect to the use of monoactivated azomethine ylides in organocatalysis were resolved due to the glycine imine activation through an intramolecular hydrogen bond (Scheme 11).^38,39^ The presence of a hydroxyl group at the *ortho* position of the aromatic ring allowed for a H-bond with the iminic nitrogen, resulting in an acidity increase of the methylene hydrogens. Thus, deprotonation with the Brønsted base of Takemoto’s catalyst generated the corresponding ylides—from aldimines and ketimines—that were subsequently added to nitroalkenes, thereby promoting [3 + 2]-cycloadditions^38^ and Michael reactions,^39^ respectively. These two reaction pathways provided access to optically pure pyrrolidines and α,γ-diamino acid derivatives in excellent results, respectively. It was found that the presence of the OH group was crucial, as the reactions in the absence of this motif inhibited the processes. In this regard, DFT calculations shed light onto the mechanism of both transformations based on Pápai–Zhong’s model to explain the coordination of the imine derivatives and the nitroalkene to the bifunctional thiourea. Moreover, an intermolecular interaction via hydrogen bonding between the *ortho*-hydroxyl group of the (ket)imines and the NH group of the thiourea motif was identified, providing additional stabilization and reactivity to the process (proposed model, purple). Despite the fact that the former work yielded pyrrolidines with four stereogenic centers, the latter case was recognized as a very interesting and attractive strategy as the ketimine was hydrolyzed and the 2-hydroxybenzophenone was recovered as a chemical auxiliary of the Michael reaction (see Scheme 11, selected derivatization).

**Scheme 11:**
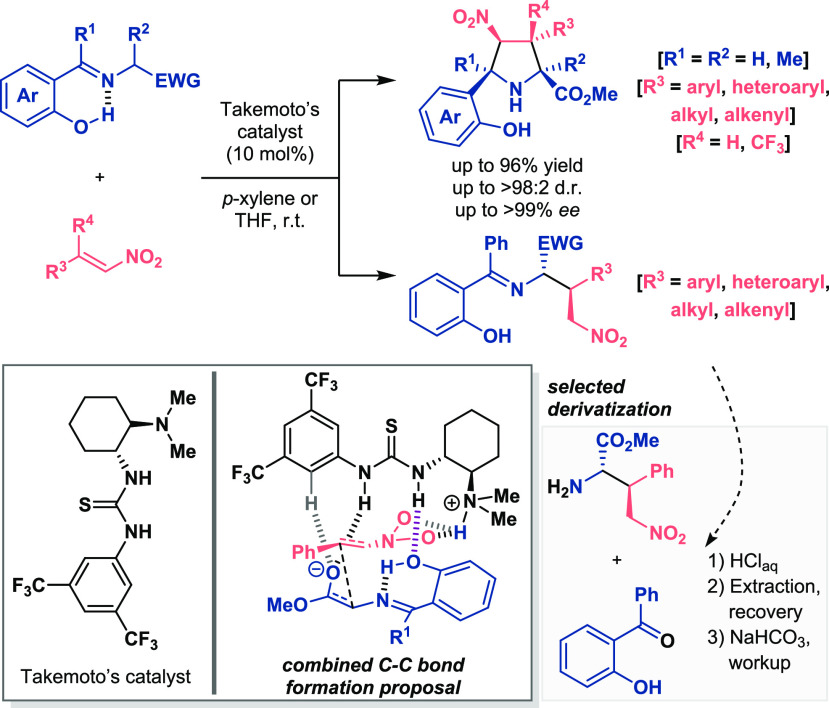
Activation of Azomethine Ylides for Enantioselective Organocatalytic [3 + 2]-Cycloaddition and Michael Addition Reactions (Alemán 2018)

The influence of the OH···N bond for the enhancement of carbon atom nucleophilicity was later extended to nitrogen. In pursuit of a more N-activated ketimine, 2-hydroxybenzophenone imine was described as a magnificent aminating reagent (Scheme 12).^40^ It was demonstrated that the hydroxyl group established a strong intramolecular H-bond with the nitrogen of the imine that increased the acidity of the NH proton. Thus, an alternative methodology for the asymmetric amination of enals using 2-hydroxybenzophenone imine derivatives as nucleophilic nitrogen sources was reported. This aminocatalytic transformation via iminium-ion activation was supported by DFT calculations that helped us to understand the reaction pathway—ketimine deprotonation, C–N bond formation, and proton transfer—and the significant role of the OH group at these stages. In addition, easy hydrolysis of the imine ended up with the synthesis of free amine-derived compounds and the recovery of the 2-hydroxybenzophenone tagged as the chemical auxiliary (see selected derivatization, Scheme 12, bottom).

**Scheme 12:**
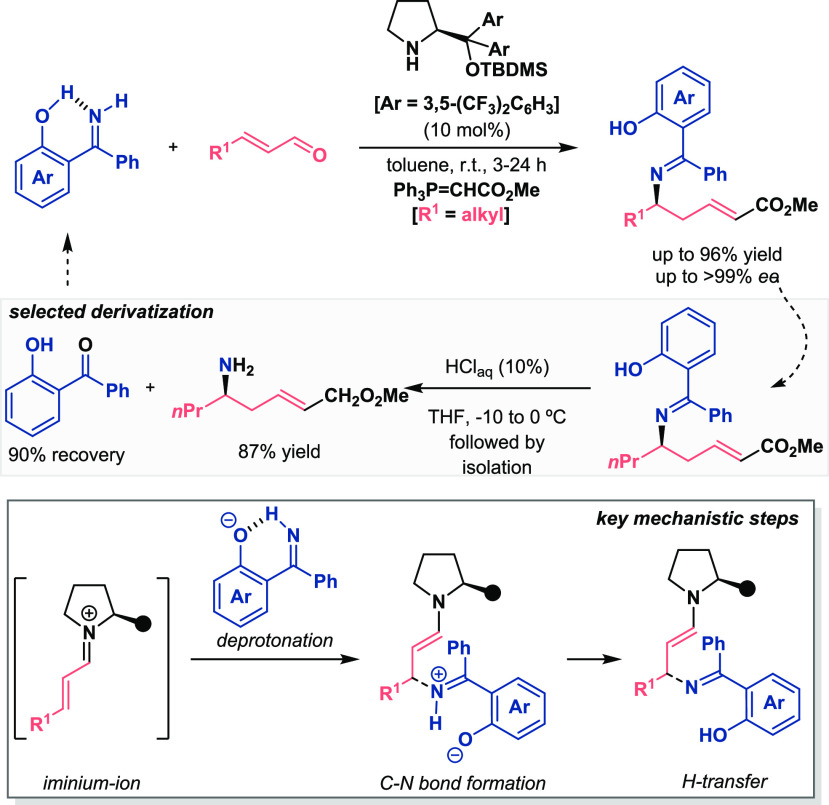
Activation of Ketimines for Enantioselective Organocatalytic Aza-Michael Addition Reaction (Alemán 2018)

Finally, the N-functionalization of nitroalkenes was envisioned in the event of an expansion of this chemical approach (Scheme 13).^41^ Given the importance of diamine derivatives in many scientific areas, the aza-Michael addition between ketimines and nitro-olefins was developed under both batch and flow conditions using a bifunctional thiourea as the catalyst. This reaction, which resulted in a limitation in the literature because moderate asymmetric induction values were obtained in the previous reported works with benzophenone imines as nucleophilic sources,^42^ succeeded as excellent yields and enantioselectivities were achieved in 20 examples. Once again, the role of the OH was verified because the aza-Michael adduct was afforded as a racemic mixture using benzophenone imine as the N-centered nucleophile. Finally, the potential of this method was shown within the hydrolysis of the imine and recovery of the starting ketone, together with the synthesis of a diamine product found as an intermediate in the preparation of a drug-like compound^11^ (see selected derivatization, Scheme 13, bottom).

**Scheme 13:**
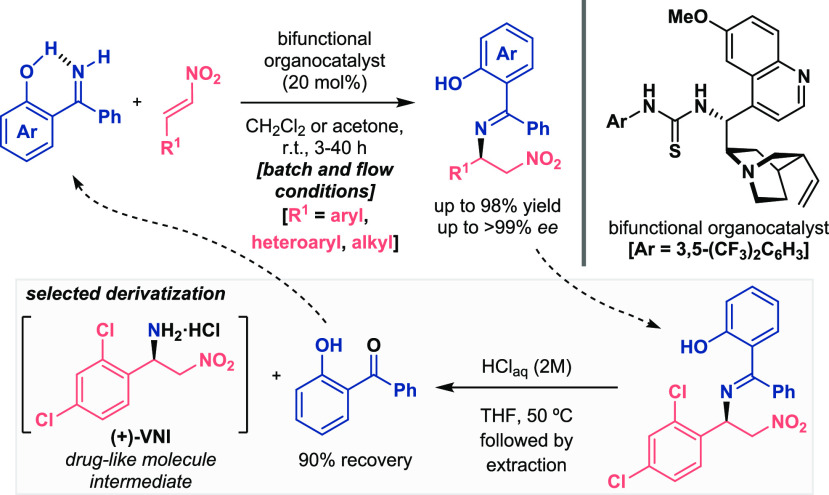
Activation of Ketimines for Enantioselective Organocatalytic Aza-Michael Addition Reaction (Alemán 2021)

In addition to enhancing the reactivity of different reagents, the intramolecular H-bond activation has featured additional roles. In this context, Zhang described the stabilization of enynamides and the control of the structure toward the asymmetric organocatalytic conjugate addition of nitromethane to afford optically pure allenamide derivatives. They claimed that the intramolecular H-bond prevented competitive reactions due to the structure arrangement and led to a proper orientation of the substrates with the catalyst.^43^ On the other hand, the presence of this interaction in the structure of Mannich-type adducts has controlled the enantioselectivity of the metal-catalyzed process between α,β-alkynyl ketones and 2*H*-azirines developed by Trost et al. (Scheme 14).^44^

**Scheme 14:**
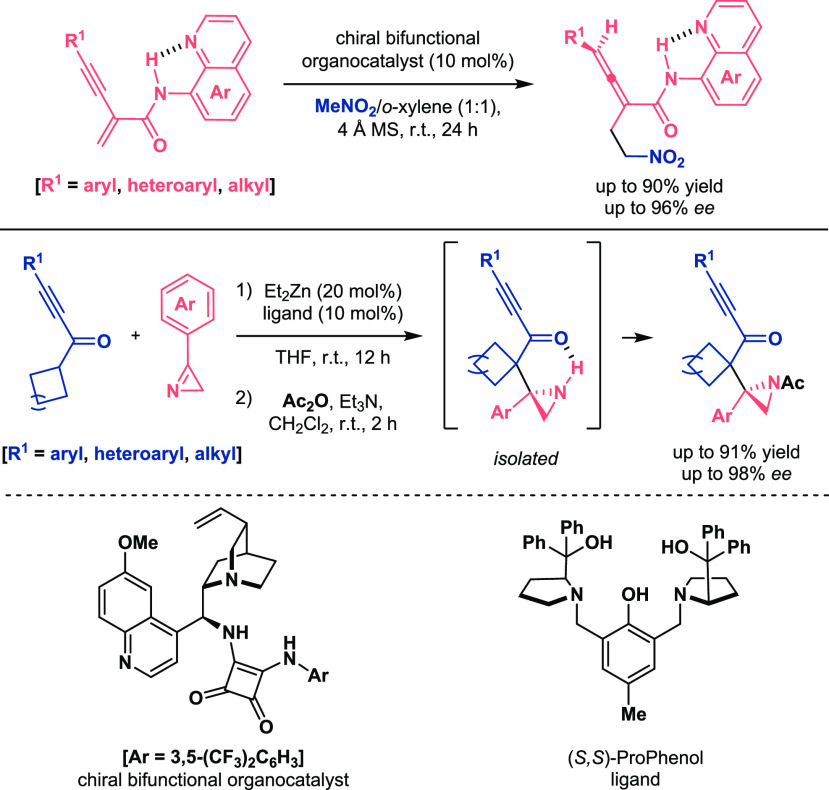
Intramolecular H-Bond Operating in Enantioselective Catalytic Michael and Mannich Reactions (Zhang 2019, Top, Trost 2020, Bottom)

Throughout this Perspective, several examples have been reviewed in which OH and NH groups have played a key role in establishing intramolecular hydrogen bonds in the substrates. This noncovalent interaction has allowed the activation of electrophiles and nucleophiles to carry out metal and organocatalytic reactions, leading to the formation of high-value products. In addition to reactivity, significant interactions with the catalytic system have proven to be key for enantiocontrol. Nonetheless, given that the presence of these functional groups can be considered as a limitation of the final compound, in some reports, those chemical functionalities have been removed or transformed in a very elegant manner. We firmly believe that the discovery of alternative structures for this purpose would be of significant interest for the scientific community, and we hope that this work could contribute to the development of novel chemical strategies to overcome present and future problems grounded on reactivity, selectivity, or even stability of the processes.
